# Navigating Healthcare AI Governance: the Comprehensive Algorithmic Oversight and Stewardship Framework for Risk and Equity

**DOI:** 10.1007/s10728-025-00537-y

**Published:** 2025-08-13

**Authors:** Rahul Kumar, Kyle Sporn, Ethan Waisberg, Joshua Ong, Phani Paladugu, Amar S Vadhera, Dylan Amiri, Alex Ngo, Ram Jagadeesan, Alireza Tavakkoli, Timothy Loftus, Andrew G Lee

**Affiliations:** 1 Department of Biochemistry and Molecular Biology, University of Miami Miller School of Medicine, Miami, USA; 2 University of Massachusetts Chan School of Medicine, Worcester, USA; 3 SUNY Upstate Medical University, Syracuse, USA; 4 University of Cambridge, Cambridge, UK; 5 Department of Ophthalmology and Visual Sciences, University of Michigan Kellogg Eye Center, Ann Arbor, USA; 6 Sidney Kimmel Medical College, Thomas Jefferson University, Philadelphia, USA; 7 Brigham and Women’s Hospital, Boston, USA; 8 University of Miami Miller School of Medicine, Miami, USA; 9 Department of Computer Science, Johns Hopkins University Whiting School of Engineering, Baltimore, USA; 10 Artificial Intelligence Systems, Cisco Inc., San Jose, USA; 11 Department of Computer Science and Engineering, University of Nevada, Reno, USA; 12 TL Insight US, Miami, USA; 13 Department of Ophthalmology, Blanton Eye Institute, Houston Methodist Hospital, Houston, USA; 14 The University of Texas MD Anderson Cancer Center, Houston, USA

## Abstract

Integrating artificial intelligence (AI) in healthcare has sparked innovation but exposed vulnerabilities in regulatory oversight. Unregulated “shadow” AI systems, operating outside formal frameworks, pose risks such as algorithmic drift, bias, and disparities. The Comprehensive Algorithmic Oversight and Stewardship (CAOS) Framework addresses these challenges, combining risk assessments, data protection, and equity-focused methodologies to ensure responsible AI implementation. This framework offers a solution to bridge oversight gaps while supporting responsible healthcare innovation. CAOS functions as both a normative governance model and a practical system design, offering a scalable framework for ethical oversight, policy development, and operational implementation of AI systems in healthcare.

**Supplementary Information:**

The online version contains supplementary material available at https://doi.org/10.1007/s10728-025-00537-y.

## Introduction

The healthcare sector’s embrace of artificial intelligence (AI) marks a critical transformation in clinical practice, yet the presence of “shadow” AI systems has become an important issue [1]. These unauthorized AI systems, which have already undergone deployment in clinical decision support, patient prioritization, and diagnostic interpretation, raise important concerns about patient equity, regulatory accountability, and safety. Without proper regulation, oversight, and validation, we risk biased decision-making, misdiagnoses, and inconsistent standards of care. This can have dramatic effects on patient quality of life and therapeutic outcomes and thus is important to correct.

Recent studies and meta-analyses have shown gaps in clinical validation among FDA-approved AI medical devices, with 43% lack of studies lacking rigorous evaluation protocols to ensure product equivalency. AI medical devices often rely on limited or biased datasets, and thus, there remains a lack of comprehensive validation in real-world healthcare settings [2]. For instance, many AI models are developed and tested on data from simple or non-representative populations, which skew their accuracy, and thus, external validity is limited across different demographics. This oversight creates significant risks, particularly when AI systems may fail to recognize subtle variations in patient conditions due to population differences. Evidence of AI-related harm continues to grow— a widely used sepsis prediction model in U.S. hospitals was found to overestimate the risk of sepsis, leading to unnecessary interventions, increased healthcare costs, and patient distress [2]. Additionally, other studies have found innate racial bias in AI-driven triage algorithms, consistently underestimating the severity of Black patients’ conditions when compared to their white counterparts, exacerbating existing healthcare disparities and potentially resulting in delayed treatments or misdiagnosis [3]. These issues emphasize the need for regulatory bodies to improve upon the existing pre-market evaluation criteria for FDA device approval and to institute better post-market surveillance systems to detect and correct failures in real time. Furthermore, shadow systems—AI algorithms operating outside of FDA oversight—are also becoming a growing concern, as they lack transparency and accountability, further complicating clinician efforts to ensure patient safety and equity in AI deployment.

As can be seen, the increasing need for fast, innovative, and regulatory effectiveness of medical devices presents unique issues in today’s healthcare landscape that need to be considered. While streamlined AI adoption protocols may accelerate technological progress [3]—particularly benefiting underfunded hospitals, resource-constrained facilities, community clinics, and emerging healthcare ventures—they also risk exacerbating disparities in quality of care. Underfunded institutions and those in medical deserts may need to use inadequately validated AI technologies, deepening existing inequities in healthcare outcomes. This discrepancy between rapid innovation and regulatory oversight necessitates a complex governance model capable of dynamically adapting to AI while preserving patient safety, transparency, and fairness.

As AI and clinical care continue to affect each other synergistically, we face a critical issue: how can we craft a governance model that evolves as quickly as the technology that it maintains, allowing for both systems to improve upon each other while safeguarding patient-protected health information (PHI) and trust in healthcare? To address these challenges, we propose the Comprehensive Algorithmic Oversight and Stewardship (CAOS) Framework, a paradigm shift in AI governance that includes:

1. Dynamic licensing systems that evolve with AI model updates to ensure continued validation.
2. Patient-centered transparency tools, such as a national registry of AI systems detailing system capabilities, limitations, and performance metrics.
3. Retroactive certification pathways for shadow AI tools demonstrating real-world safety and efficacy, enabling responsible integration into clinical workflows.

This article explores these solutions and their ethical implications, offering a roadmap for a future where innovation drives equity and accountability in AI-driven healthcare. Our approach reflects an understanding of the distribution of governing authority across executive, legislative, and judicial branches when discussing potential regulatory approaches, emphasizing the need for a coordinated regulatory response to the growing influence of AI in medicine. Though each CAOS component can be adopted individually, the framework is designed as an integrated ecosystem: Stratified Algorithmic Risk Assessment Protocol’s (SARAP) risk tiering informs the retraining cadence under Algorithmic Bias and Equity Mitigation Framework (ABEMF), the interpretability thresholds under Causal Inference and Counterfactual Reasoning Engine (CICRE), and the regulatory responsiveness in Dynamic Regulatory Adaptation Mechanism (DRAM). Grounded in principles of equity, accountability, and patient safety, the CAOS Framework embodies a normative commitment to just and transparent healthcare innovation.

### The Problem of Shadow AI in Healthcare

Recent developments in healthcare AI, such as Stanford’s deployment of DAX Copilot for clinical documentation [4], highlight growing concerns about unregulated AI tools in medical settings. While the FDA provides oversight through the Federal Food, Drug, and Cosmetic Act (FDCA) and the Software as a Medical Device (SaMD) framework, these regulations focus primarily on static, pre-market evaluations and lack mechanisms to effectively regulate self-learning AI systems [5]. The 21st Century Cures Act further establishes pathways for digital health innovations but excludes certain clinical decision support tools from FDA regulation [7, 8, 21], creating additional oversight gaps. As AI increasingly influences clinical decisions, traditional legal doctrines focused on point-of-manufacture defects prove insufficient, while conflicting state and federal laws, such as the California Consumer Privacy Act (CCPA) and HIPAA, allow shadow AI developers to exploit regulatory inconsistencies [9, 10]. Real-world examples reinforce these concerns: Epic Systems’ widely deployed sepsis prediction algorithm, used in over 100 U.S. hospitals, was found to miss two-thirds of actual sepsis cases while frequently issuing false positives, reflecting a lack of independent validation and transparency [70]. In the mental health domain, unvalidated AI chatbots have similarly raised ethical alarms. The UK’s National Health Service adopted tools like Limbic for mental health triage without peer-reviewed testing, and a disturbing case involving the Character.ai chatbot allegedly encouraging self-harm in a teenager underscores the psychological dangers of unregulated, unsupervised AI use [71].

Within the broader CAOS Framework, the AI Liability Certification (AILC) establishes rigorous protocols for testing AI systems against diverse patient populations and real-world clinical scenarios. Our approach aims to successfully fill the gaps in existing oversight, particularly those left open by discrepancies in current regulatory frameworks, including the 21st Century Cures Act. Although not one of the seven core components, AILC functions as a cross-cutting sub-framework that draws on outputs from these modules—particularly SARAP risk tiering, CICRE interpretability reports, and ABEMF fairness audits—to generate a formal certification dossier. In this way, AILC operationalizes the framework’s accountability mechanisms by translating ongoing monitoring and ethical evaluations into a unified liability and safety validation structure for both pre- and post-deployment settings.

A key challenge in AI oversight is performance degradation, particularly when AI systems autonomously adjust their decision-making protocols [6]. For instance, an AI tool for detecting diabetic retinopathy may lose accuracy as patient demographics shift, yet current post-market surveillance mechanisms, which monitor devices based on original specifications, are inadequate for capturing such real-time performance changes. For example, the Epic Sepsis Model, deployed in numerous U.S. hospitals, experienced significant performance drift during the COVID-19 pandemic. As patient demographics and healthcare practices shifted rapidly, the model’s accuracy declined, leading to an increase in false positives and unnecessary clinical alerts [72]. This case underscores the inadequacy of current post-market surveillance mechanisms, which often fail to capture real-time performance changes in adaptive AI systems.

Additionally, the “shadow” AI platforms that exist without formal authority pose significant clinical data leaks of PHI. While the 2017 WannaCry cyberattack on the UK’s National Health Service was not directly caused by AI, it underscored the broader vulnerability of healthcare systems to cyber threats [11]. The incorporation of shadow AI platforms-unregulated tools lacking formal authorization-into such networks can exacerbate these risks, further compromising patient data and essential medical services. Unlike WannaCry, real-world instances of shadow AI have directly led to serious consequences, reinforcing the urgent need for robust governance and oversight [73]. For instance, Babylon Health’s AI chatbot, deployed for medical triage, was criticized for providing potentially unsafe advice, including misdiagnosing critical conditions [73]. The UK’s Medicines and Healthcare products Regulatory Agency (MHRA) raised safety concerns, underscoring the lack of regulatory safeguards for such tools [74]. In another case, researchers at the University of Oxford cautioned against the use of generative AI platforms like ChatGPT and Bard to create social care plans, warning that these systems could compromise patient confidentiality and deliver substandard outputs. As Dr. Caroline Green noted, inputting sensitive data into these models may lead to unintended use in training datasets, raising serious ethical and legal concerns [75]. These cases highlight how the unchecked integration of shadow AI tools can jeopardize both patient safety and data integrity, reinforcing the urgent need for comprehensive governance frameworks to detect, regulate, and retroactively certify such systems.

Our proposed CAOS framework is designed to bridge these regulatory gaps by incorporating adaptive, risk-based oversight mechanisms that ensure ongoing safety and efficacy beyond initial approval. Unlike current static models that rely on pre-market evaluations and frequent reassessments, the CAOS framework introduces a structured post-market monitoring system that dynamically integrates real-world performance clinical data to refine regulatory decisions. By addressing these deficiencies, CAOS offers a more comprehensive and responsive regulatory paradigm for AI-driven healthcare technologies. To address these challenges, we urgently need a comprehensive, adaptive governance model that:

1. Addresses the evolving nature of AI systems.
2. Harmonizes conflicting regulations.
3. Establishes clear accountability mechanisms.
4. Ensures equitable deployment and performance across diverse patient populations.

This new framework would govern AI in healthcare, but it would still require a delicate balance between fostering medical advancement and maintaining strict oversight to protect patients and preserve confidence in AI-enabled healthcare delivery.

### The CAOS Framework for Healthcare AI Implementation

Our proposed CAOS Framework tackles these intricate challenges through an integrated approach, incorporating input from clinicians, patients, insurers, and oversight agencies.

A key enhancement within CAOS is the integration of the System for Adaptive Risk Assessment and Prediction (SARAP) [12] and the Dynamic Regulatory Adjustment Mechanism (DRAM). SARAP proactively tracks shifts in AI behavior, resolving risks that could emerge before they could even compromise patient safety—an improvement over existing regulatory frameworks that rely on periodic or event-driven reassessments that would only resolve problems after the fact. On the other hand, DRAM enables real-time regulatory adjustments based on continuous performance metrics, affording a more flexible and dynamically responsive oversight model. In contrast, the FDA’s current risk classification is static and cannot accommodate rapidly evolving AI behaviors without requiring formal reclassification or additional regulatory submissions.

**Fig. 1:**
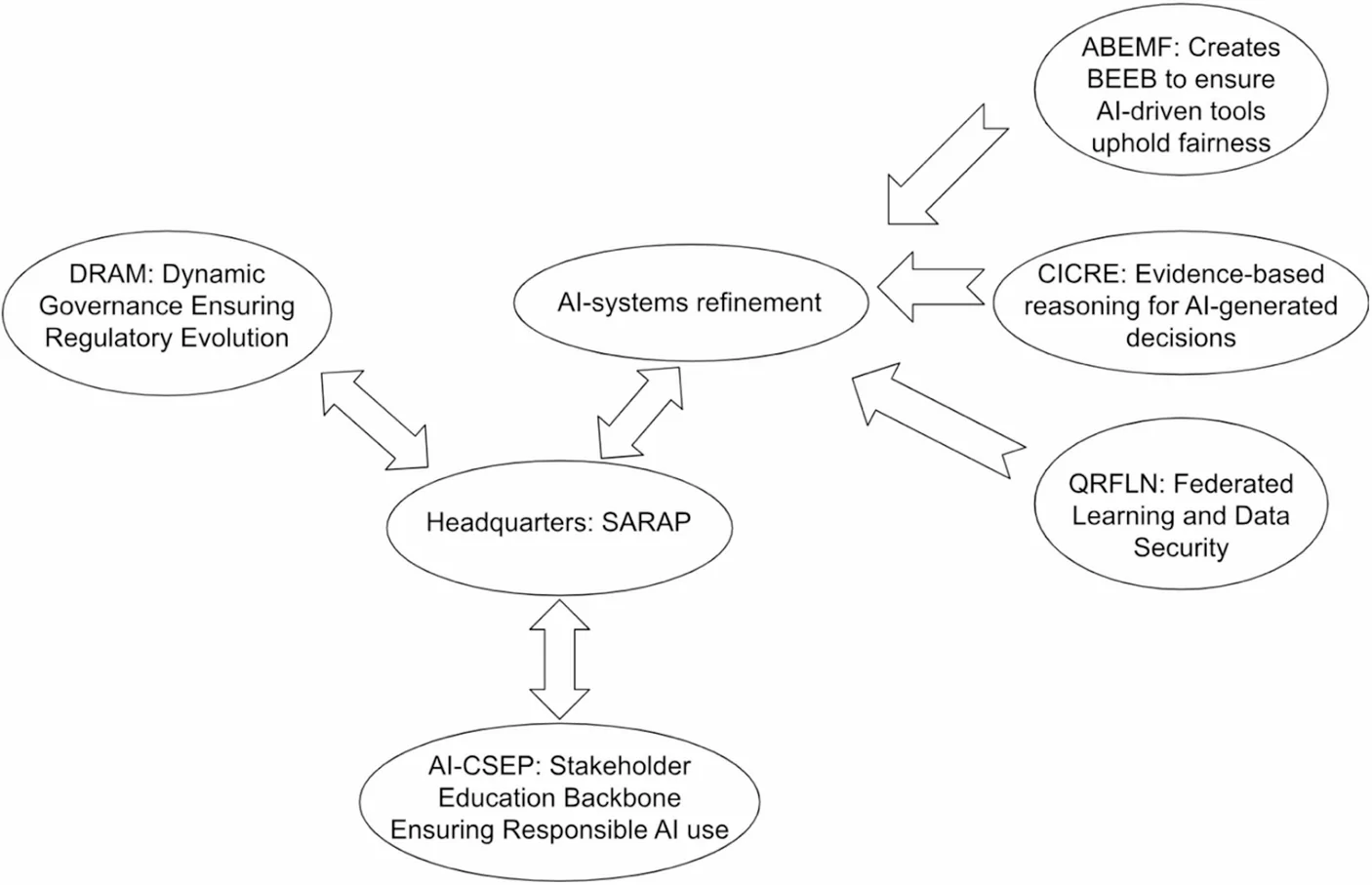
A structured diagram illustrating how the components of the CAOS Framework interact with each other and existing regulatory mechanisms

CAOS thus presents a prospective regulatory model that enhances patient safety while fostering responsible innovation. Its continuous monitoring and dynamic risk stratification mechanisms ensure that AI-driven medical technologies remain effective, accountable, and adaptable throughout their lifecycle (Fig. 1).

## Stratified Algorithmic Risk Assessment Protocol (SARAP)

SARAP forms the foundation of the CAOS framework, providing a complex, multi-layered methodology for evaluating AI systems according to their clinical significance, data privacy requirements, and operational importance [43, 44]. These assessments would be conducted by designated AI oversight committees within hospital regulatory boards or national health agencies; assessments would be conducted by designated AI oversight committees within hospital regulatory boards or national health agencies to ensure consistent and objective risk evaluations across healthcare institutions, standardizing evaluation criteria, enforcing uniform assessment protocols, and leveraging external audits to maintain cross-institutional consistency [44, 45].

SARAP uses a three-dimensional risk evaluation system that examines Clinical Impact (CI), Data Sensitivity (DS), and Operational Dependency (OD), each quantified on a continuous scale from 0 to 1 to enable precise risk stratification [12]. This system ensures that AI systems are evaluated with clarity and technical accuracy, use conversational language patterns, and preserve rigorous evaluation standards [46].

The Clinical Impact (CI) dimension assesses the direct effect of the AI system on patient care decisions, ranging from low-risk administrative tasks with no direct patient impact ((e.g., scheduling software) to high-risk, life-critical interventions (e.g., AI-powered ICU decision support). Real-world validation studies reinforce this classification, demonstrating how predictive models such as Score for Emergency Risk Prediction (SERP) impact mortality risk stratification in emergency departments, with AUC scores ranging from 0.874 to 0.905. These studies highlight the challenges of clinical integration, emphasizing that the use of AI in the clinical setting can dramatically change a patient’s therapeutic outcomes [46, 47].

Data Sensitivity (DS) is evaluated using a modified version of the Caldicott Principles [13], considering factors such as the type and volume of protected health information (PHI) processed to ensure patient privacy and compliance. These principles align with risk-based monitoring (RBM) methods in clinical trials, which have successfully minimized efficacy-related errors (0.15%) and safety data errors (0.49%). SARAP’s DS criteria mirror these guidelines by prioritizing minimized data use, justified processing purposes, and compliance with privacy regulations [48].

Operational Dependency (OD) measures the extent to which critical healthcare infrastructure relies on the AI system, from standalone tools to fully integrated hospital networks. Studies support OD evaluation through AI integration methodologies, including edge device deployment and automated risk detection [49]. There remain practical challenges in implementing SERP in clinical practices, such as in balancing AI-generated decisions with clinical workflows and mitigating alarm fatigue that emphasize the importance of seamlessly integrating these technologies while minimizing disruption of existing healthcare operations [46].

This structured approach provides a detailed, scalable framework for evaluating AI systems in healthcare, ensuring that risk assessments are both comprehensive and adaptable across diverse clinical environments. The resulting three-dimensional risk score (R), **Eq. 1**, is calculated using a normalized Euclidean distance formula [14], where CI is Clinical Impact, DS is Data Sensitivity, and OD is Operational Dependency:

$$\:R=\sqrt{{\left(CI\right)}^{2}+{\left(DS\right)}^{2}+{\left(OD\right)}^{2}}$$

### Equation 1

Three-dimensional risk score (R) equation.

This approach ensures that the risk score comprehensively reflects the multifaceted nature of AI deployment in healthcare settings. Based on this score, AI systems are classified into five tiers, each associated with progressively stringent oversight mechanisms [50–52]:

- Tier 0 (Minimal Risk) (*R* < 0.2): AI tools with negligible patient impact, such as automated appointment reminders.
- Tier 1 (Low Risk) (0.2 ≤ *R* < 0.4): AI models assisting with non-critical clinical decisions, like natural language processing for medical transcription.
- Tier 2 (Moderate Risk) (0.4 ≤ *R* < 0.6): AI applications that influence diagnostic processes but do not directly determine treatment, such as AI-assisted radiology screening.
- Tier 3 (High Risk) (0.6 ≤ *R* < 0.8): AI tools directly impact patient management, like predictive analytics for ICU deterioration risk.
- Tier 4 (Critical Risk) (*R* ≥ 0.8): AI models functioning as autonomous or semi-autonomous decision-makers, including robotic surgical assistants or AI-driven sepsis intervention systems.

The tiered approach within the risk-tiering system —ranging from Tier 0 (Low Risk) to Tier 4 (Critical Risk) — reflects ethical principles such as proportionality and non-maleficence, ensuring that the level of scrutiny aligns with the potential for patient harm and system disruption. For instance, Tier 4 includes autonomous AI systems involved in critical clinical decisions, demanding the highest level of scrutiny to protect patient safety and uphold ethical standards. This ethical alignment complements the FDA’s risk-based credibility framework, which evaluates “model influence” and “decision consequence” to determine oversight levels—paralleling the *R* ≥ 0.8 threshold for Tier 3 and Tier 4 classification [53]. The NIST AI Risk Management Framework (AI RMF) further supports this structure, scaling governance functions (GOVERN, MAP, MEASURE, MANAGE) with risk tiers. Higher-tier AI systems require enhanced transparency, including explainability requirements for Tier 4 models [53, 54].

Importantly, SARAP directly informs other components of the CAOS Framework. For example, ABEMF’s Equity Calibration Pipelines are triggered by the risk-tiering system’s designations: Tier 3 and Tier 4 systems must undergo more frequent equity audits and retraining intervals due to their higher clinical stakes and broader impact on diverse patient populations. Likewise, CICRE’s interpretability protocols scale with SARAP classification—lower-tier systems may require only basic model explainability, while Tier 4 systems must integrate full causal reasoning and counterfactual transparency to support epistemic accountability in critical care settings. These tier-dependent requirements reinforce the cohesion of the CAOS Framework, ensuring each governance layer is both risk-sensitive and ethically responsive.

Tracking shifts in AI behavior involves monitoring performance over time using benchmark datasets, continuous validation cycles, and algorithmic audit logs to detect changes in sensitivity, specificity, or fairness. The tiered approach is validated by AI risk management frameworks, which emphasize risk prioritization based on projected clinical impact. Real-world examples demonstrate the implementation of high-risk AI (Tier 3/4) in chronic disease management and ICU predictions, highlighting the clinical relevance of structured oversight [55]. Additionally, resource-intensive “MANAGE” protocols ensure that critical-risk AI systems undergo rigorous evaluation, documentation, and post-deployment monitoring [53]. To address the dynamic nature of AI performance, the risk-tiering system incorporates continuous monitoring mechanisms that assess real-time performance metrics, including accuracy, calibration, and fairness across diverse patient populations. This ongoing evaluation is essential for detecting shifts in AI behavior—such as performance degradation or unintended biases—that may emerge due to evolving input data or clinical practices. By integrating these monitoring processes, SARAP ensures that AI systems maintain their reliability and effectiveness over time, thereby mitigating risks associated with “shadow AI”—unregulated or unauthorized AI tools operating without formal oversight.

By incorporating tier-specific governance measures and continuous evaluation processes, this framework ensures that regulatory resources are allocated efficiently, applying the highest scrutiny to AI systems with the greatest potential risk while balancing innovation with patient safety in healthcare applications. The framework’s emphasis on transparency and accountability not only safeguards against potential data breaches and clinical errors but also reinforces trust in AI applications within healthcare settings.

## Quantum-Resistant Federated Learning Network (QRFLN)

The CAOS framework also requires the implementation of an advanced Quantum-Resistant Federated Learning Network (QRFLN) across all Tier 2 and higher AI platforms, answering concerns over data security and model efficacy in medical AI applications [15]. Beyond mere legal compliance, QRFLNs operationalize normative commitments to privacy, data sovereignty, and patient control by embedding privacy-preserving technologies that empower institutions and patients alike. QRFLNs employ a lattice-based cryptography to secure federated learning against potential computing attacks, allowing for geographically distant healthcare institutions to collaboratively train and share AI models with each other without sharing raw patient data. This ensures compliance with HIPAA’s data minimization principles while improving AI model generalizability [16].

The key to the explainability and consent framework’s improvement over current systems is its Homomorphic Encryption Layer, which allows for computations on encrypted data and model updates without ever revealing sensitive information, thereby operationalizing a trust architecture that shifts power toward data custodians and patients rather than centralized entities. The system uses sophisticated privacy-enhancing techniques that introduce carefully calibrated statistical noise during model updates, effectively preventing patient identification while maintaining data integrity [17]. The framework integrates with advanced Multi-Party Computation (MPC) protocols to allow for secure, decentralized collaboration among healthcare institutions while protecting proprietary contributions, thereby fostering innovation in medical AI development that balances openness with intellectual property rights.

However, implementing QRFLN presents challenges for smaller healthcare organizations with limited technical expertise and outdated infrastructure. Many facilities rely on outdated health information systems that would require many hardware changes before being compatible with the explainability and consent framework’s advanced cryptographic and federated learning protocols [56]. These concerns need to be addressed with low-cost alternatives or transition pathways to accommodate the various technical capacities of healthcare institutions and align with CAOS’s commitment to equitable AI governance.

QRFLNs need to be implemented using cost-effective, scalable solutions. For example, lightweight lattice-based encryption provides an affordable alternative to costly quantum-resistant cryptographic hardware. Resource-limited hospitals can use open-source cryptographic libraries such as CRYSTALS-Kyber, a NIST-standardized post-quantum cryptographic algorithm, to enable secure AI model training without requiring major hardware upgrades [22]. Additionally, federated learning with regional nodes offers a reliable solution by allowing smaller hospitals to contribute to AI development through centralized hubs, like university hospitals, which manage most of the model training and democratize participation while preserving privacy [23]. Edge computing devices like NVIDIA Jetson Nano or Raspberry Pi can be equipped with encryption modules to ensure privacy-preserving federated learning at the point of care without significant infrastructure investment [24, 25].

Recognizing the current technological and infrastructural constraints, the CAOS framework pragmatically incorporates alternative strategies that complement QRFLNs, thereby ensuring broader adoption without compromising ethical standards or compliance. Pre-trained AI models from larger healthcare networks can be fine-tuned by low-resource hospitals, reducing the computational burden of training models from scratch and facilitating localized adaptation of AI tools. Cloud-based AI frameworks, such as Google Cloud’s Federated Learning API or Microsoft Azure’s Confidential Computing, offer scalable, secure infrastructures that can be easily implemented and replace the need to install high-cost new systems while maintaining compliance with privacy and security mandates [24–27]. These approaches not only enhance accessibility but also operationalize CAOS’s normative commitment to data sovereignty by enabling institutions to retain control over their data contributions and AI model adaptations.

Overall, QRFLNs and these complementary solutions collectively uphold the ethical pillars of the CAOS framework—privacy, transparency, accountability, and equitable participation —while aligning with formal institutions such as HIPAA, FDA, and the 21st Century Cures Act by ensuring that federated learning does not involve raw patient data sharing. By integrating quantum-resistant security, scalable encryption, and a decentralized learning approach, the framework provides a solution for secure, collaborative AI development in healthcare. Addressing implementation challenges through scalable transition pathways also ensures that even resource-limited healthcare organizations can adopt the explainability and consent framework without compromising patient data security or compliance with evolving AI governance policies [26–28].

## Causal Inference and Counterfactual Reasoning Engine (CICRE)

The Causal Inference and Counterfactual Reasoning Engine (CICRE) component enhances the CAOS framework by delivering transparent, evidence-based reasoning for AI-generated decisions in Tier 3 and Tier 4 systems, addressing the increasing demand for interpretable healthcare AI. CICRE uses advanced statistical modeling and mathematical frameworks to decode AI decision-making pathways that are grounded in causal inference principles. This methodology enables the clinician to use the software to understand how the AI system reached their diagnostic or therapeutic suggestions and how the patient would respond under different therapeutic outcomes. Such interpretability is not merely technical—it reinforces epistemic accountability by making the model’s underlying logic, assumptions, and uncertainties visible to human decision-makers. This is especially critical in high-risk clinical contexts, where opaque or unchallengeable AI decisions can erode clinician confidence or lead to poor outcomes. By explicitly surfacing causal pathways and offering testable, alternative outcomes, this AI output explainability system allows clinicians to engage meaningfully with the AI’s reasoning process, improving shared decision-making and aligning with ethical standards for explainable medical technologies. The level of explanation generated by CICRE is calibrated according to the SARAP-assigned risk tier. For example, Tier 4 systems demand counterfactual transparency and full causal modeling to satisfy the interpretability standards necessary for high-risk clinical environments. As a result, CICRE can build trust among medical professionals in high-risk clinical scenarios while aligning with the FDA’s Software as a Medical Device (SaMD) guideline, which mandates AI interpretability for regulatory approval [39, 40].

A critical consideration is whether this AI output explainability system requires redesigning existing AI frameworks or can be seamlessly integrated into current infrastructures. Given its modular design, CICRE can be retrofitted into existing AI-driven clinical workflows, working in tandem with electronic health records (EHR) platforms and decision-support systems without necessitating a complete overhaul. This adaptability ensures that hospitals with varying AI maturity levels can implement an explainability system without major infrastructure changes.

To ensure robust validation of AI-generated explanations, CICRE’s causal inference models must undergo rigorous clinical validation. A simulated deployment using historical patient data from open-access medical datasets such as MIMIC-III ICU data can serve as a baseline for evaluating accuracy [34–37]. MIMIC-III contains granular ICU patient records (including vital signs, lab results, and medications) and has been widely used in clinical AI research. Running CICRE on real patient records would demonstrate how causal inference improves decision-making in settings with variable data quality, providing empirical evidence of effectiveness [31, 38].

To accommodate hospitals with limited computational resources, an AI explainability system supports efficient, resource-conscious implementation strategies. One such strategy involves simple Python libraries like DoWhy and EconML that use minimal computational power and can be easily implemented on standard hospital servers without the need for GPUs [29–31]. Another option for hospitals lacking centralized AI servers is edge-based causal AI devices, such as Medtronic’s FDA-cleared GI Genius System, which can execute pre-compiled causal inference algorithms with minimal processing demands [32]. For example, an on-device CICRE implementation could analyze AI-driven radiology predictions, compare them to historical patient data, and generate counterfactual alerts when biases are detected [33].

By integrating lightweight causal inference models like Python libraries and edge-based processing like the GI Genius System, AI explainability systems can ensure that even resource-limited healthcare settings can benefit from interpretable AI without requiring costly new infrastructure investments. Furthermore, its efficient computational design prevents unnecessary performance bottlenecks, ensuring seamless integration into existing clinical workflows while maintaining real-time AI interpretability.

## Algorithmic Bias and Equity Mitigation Framework (ABEMF)

The Algorithmic Bias and Equity Mitigation Framework (ABEMF) extends the CAOS Framework to operationalize continuous equity monitoring and proactive bias mitigation. Building upon the fairness measures within CICRE, ABEMF mandates the creation of a centralized Bias and Equity Evaluation Board (BEEB) to oversee compliance across all tiers of AI systems. This board ensures that AI-driven clinical decision-support tools uphold fairness, transparency, and accountability, reducing the risk of systemic biases in healthcare AI applications. The bias mitigation pipeline is guided by a composite normative approach to fairness, integrating principles from multiple philosophical models. Specifically, the framework draws on demographic parity to ensure equal algorithmic treatment across groups [76], procedural justice to guarantee transparency and inclusivity in audit and retraining processes [77, 78], and substantive outcome equity to reduce health disparities in clinical decision-making [77, 79]. These normative commitments are reflected in the explicit use of fairness metrics such as Demographic Parity Difference (DPD) [76], Equalized Odds Ratio (EOR) [80], and Intersectional Fairness Score (IFS) [79], and reinforced through ABEMF’s auditing and retraining mechanisms.

To maintain long-term fairness, the bias mitigation pipeline also introduces Equity Calibration Pipelines, requiring all SARAP Tier 3 and Tier 4 AI systems to undergo equity-specific retraining every six months using diverse, real-world data to maintain long-term fairness across several demographics. However, an adaptive retraining approach-based algorithm performance drift, demographic data shifts, and real-world clinical feedback may be more effective. Systems with stable bias metrics could have extended retraining intervals, while those exhibiting significant equity deviations would require more frequent updates.

This adaptive retraining approach aligns with MLOps strategies, where model retraining is triggered by data/concept drift detection [57, 58]. Defining degradation thresholds through offline experiments and balancing retraining frequency with operational costs ensures both efficiency and fairness. Additionally, Deep Reinforcement Learning (DRL) with attention mechanisms enables real-time feature prioritization adjustments, combating data drift seen in evolving clinical patterns, like fraud detection adapting to seasonal changes [59].

Pre-deployment equity audits are mandated and reviewed by regulatory agencies like the FDA, requiring developers to submit detailed impact analyses that quantify potential disparities in outcomes across demographic subgroups [18]. The audits follow Resolution Economics’ three-phase approach [60], which includes pre-implementation, pilot studies, and post-implementation phases. During the pre-implementation phase, a simulated impact analysis is conducted using custom datasets. Pilot studies involve controlled testing before full deployment, while the post-implementation phase focuses on continuous monitoring of real-world AI usage data.

These audits use explicit metrics, such as the Demographic Parity Difference (DPD), which measures differences in AI recommendations across groups; the Equalized Odds Ratio (EOR), which ensures AI models have consistent sensitivity and specificity across demographics; and the Intersectional Fairness Score (IFS), which evaluates bias across multiple demographic factors, including race, gender, and socioeconomic status [60]. Regulatory agencies review these audits to enforce compliance and ensure that AI models meet predefined fairness benchmarks before deployment.

A significant challenge in equity-focused retraining is the availability and quality of diverse, real-world data, especially for historically marginalized populations. To address this issue, ABEMF establishes Data Augmentation and Bias Correction Strategies. One of these strategies is synthetic data generation, which involves creating representative datasets using techniques like Generative Adversarial Networks (GANs) to supplement underrepresented groups [61]. Another approach is reweighting and resampling, which adjusts training datasets to reduce the overrepresentation of majority populations while preserving clinical validity [62]. Additionally, community-engaged data collection plays a crucial role, as ABEMF partners with hospitals, public health organizations, and advocacy groups to enhance data diversity and improve cultural competency in AI training [63].

To ensure real-world accountability, the bias mitigation pipeline uses algorithmic feedback portals within healthcare systems that provide accessible reporting mechanisms for patients, providers, and community stakeholders to flag potential biases in AI-generated outputs. Feedback is continuously integrated into Equity Calibration Pipelines, ensuring that AI models evolve based on real-world bias detection. To support under-resourced institutions, these pipelines are designed for modular deployment using scalable bias audit templates, allowing organizations to prioritize core fairness assessments based on their data availability and infrastructure capacity. Additionally, the ABEMF enforces Intersectional Risk Weighting (IRW), which requires algorithms to implement mathematical constraints that penalize outcomes disproportionately impacting vulnerable populations. This ensures that equity remains a core performance metric rather than a secondary consideration.

## Dynamic Regulatory Adaptation Mechanism (DRAM)

The Dynamic Regulatory Adaptation Mechanism (DRAM) introduces an agile governance model to rapidly adapt to emerging AI technologies and their associated risks, complementing the stratified oversight and real-time monitoring processes of SARAP, ensuring a proactive regulatory approach.

To enhance regulatory responsiveness and evaluate new technologies, DRAM implements Multi-Stakeholder Policy Roundtables (MPRs), convening quarterly roundtables comprising regulators, AI developers, clinicians, and patient advocates. These roundtables aim to facilitate collaborative decision-making and ensure that diverse perspectives inform regulatory strategies.

To ensure consistent assessment protocols, the dynamic regulatory update mechanism mandates the use of standardized evaluation frameworks, such as structured risk-scoring models and Rapid Ethical Impact Assessments (REIAs), to guide regulatory recommendations, technology updates, and deploy machine learning tools to predict potential ethical challenges and using historical AI deployment data to identify patterns [19].

However, the implementation of such adaptive governance faces practical challenges, particularly within regulatory institutions that are often slow-moving and fragmented. To address these constraints, DRAM proposes embedding policy prototyping and stakeholder deliberation units within existing agencies, enabling focused, iterative governance without requiring a complete institutional overhaul. Additionally, the frequency and scope of evaluations are stratified according to AI system risk level—Tier 3 and Tier 4 systems may require quarterly reporting, while lower-risk systems could follow biannual or annual review cycles. This allows the dynamic regulatory update mechanism to remain both responsive and feasible across a spectrum of healthcare environments.

To further support performance monitoring, developers of high-risk AI systems would be required to submit concise quarterly performance summaries, including key metrics such as calibration drift, accuracy degradation, and known demographic disparities. For continually learning models, this may include securely sharing model checkpoints or change logs without exposing proprietary weights. These measures help ensure that DRAM not only supports agile regulation but also directly addresses the risks posed by “shadow AI”—unregulated or unauthorized tools deployed without accountability. By enforcing structured reporting and integrating this data into the regulatory roundtable process, the dynamic regulatory update mechanism helps bring untracked AI systems under formal oversight, enabling retroactive review and certification.

Recognizing the potential for stakeholder disagreements in policy development, DRAM incorporates a Consensus Resolution Framework (CRF) to facilitate effective decision-making. This framework includes Mediation Processes led by neutral third-party ethics committees to resolve disputes impartially. Additionally, Weighted Decision Modeling ensures that stakeholder input is prioritized based on expertise and impact analysis, balancing diverse perspectives. To maintain flexibility, Iterative Policy Prototyping allows for incremental regulatory adjustments rather than rigid binary approvals or rejections, enabling a more adaptive and inclusive approach to AI governance [64].

Furthermore, DRAM establishes a Global Alignment Network (GAN) to create international partnerships for harmonizing AI regulations and creating a shared repository of best practices, regulatory guidelines, and failure case studies. GAN facilitates regulatory harmonization workshops, where nations collaborate to develop shared compliance standards based on empirical AI deployment data. These workshops contribute to the creation of a unified best practices repository, which compiles regulatory guidelines, failure case studies, and audit methodologies to ensure cross-border alignment. Additionally, GAN conducts interoperability stress testing, evaluating AI systems across different regulatory landscapes to ensure they comply with diverse regulations and can adapt to various requirements [65]. The dynamic regulatory update mechanism dynamically prioritizes policy responses and regulatory updates using structured inputs from SARAP risk scores, CICRE-derived transparency metrics, and ABEMF audit data, enabling timely, evidence-informed oversight that adapts to evolving AI performance.

By integrating structured assessment protocols, conflict resolution mechanisms, stratified reporting requirements, and global harmonization strategies, DRAM fosters a cohesive, responsive, and internationally aligned AI governance model. Most importantly, its emphasis on adaptive oversight directly responds to the evolving risks associated with both regulated and shadow AI systems, helping protect patient safety and uphold ethical standards in real-world healthcare deployment.

## AI Competency and Stakeholder Education Program (AI-CSEP)

To ensure effective AI oversight in healthcare, we propose the AI Competency and Stakeholder Education Program (AI-CSEP), designed to enhance AI literacy, ethics, and governance across all levels of healthcare interaction. The program includes a Healthcare Professional Certification (HPC) with tiered certification programs, ensuring training depth aligns with system interaction levels and associated risk classifications. HPC is required for clinicians using AI systems, with scalability addressed through a modular, role-based curriculum. This allows lower-risk users to complete streamlined courses while high-risk AI users undergo more in-depth training.

AI-CSEP also mandates the implementation of Institutional AI Readiness Programs within healthcare organizations, providing structured training modules for administrators and IT teams managing AI deployments [20]. Additionally, the program creates Patient Education Portals (PEPs), offering accessible explanations of AI processes, risks, and benefits, thereby empowering patients to make informed decisions about their care.

To validate ongoing competency, the AI literacy and training module introduces a blockchain-based credentialing system for Continuous Competency Tracking, ensuring secure, tamper-proof documentation of AI education [66, 67]. Blockchain is chosen for its decentralized verification, interoperability across institutions, and resistance to data tampering, providing a transparent and scalable solution for tracking evolving AI expertise [66–68].

Blockchain is chosen not only for its technical resilience—such as decentralized verification and resistance to data tampering—but also for its ethical advantage in supporting transparent, trust-enhancing credentialing across institutional boundaries [81–83]. While traditional centralized databases can provide basic audit trails, they often lack interoperability and are more vulnerable to manipulation or administrative opacity [81]. Blockchain offers a uniquely decentralized and ethically aligned infrastructure for healthcare credentialing by enabling cross-institutional verification without reliance on a single gatekeeping entity [84]. Its use in AI-CSEP underscores a commitment to accountability, verifiability, and patient trust in clinician competence, particularly in high-risk AI-assisted care contexts [81–84].

## Equity-Centric AI Implementation Protocol (EAIP)

The Equity-Centric AI Implementation Protocol (EAIP) extends the CAOS Framework to address systemic healthcare inequities exacerbated by unregulated AI deployment. EAIP requires annual Regional Equity Audits (REA) to assess AI system performance in underserved areas, with public reporting to promote transparency. These audits employ specific methodologies such as collecting demographic and socioeconomic data, conducting stakeholder interviews, and performing algorithmic impact assessments across diverse healthcare settings. Studies can assess equity impact using measures like healthcare access, health outcomes, and patient satisfaction across different population groups, ensuring a comprehensive understanding of the AI system’s effect.

The protocol introduces Resource Allocation Incentives, offering federal grants for AI developers incorporating equity-by-design principles into their algorithms, encouraging innovation while addressing disparities. The ethical deployment protocol also establishes Community-Driven Algorithmic Design. To increase feasibility in under-resourced regions, the ethical deployment protocols can be adapted through low-cost community engagement methods, reliance on open-source equity audit tools, and integration with regional data hubs rather than institution-specific infrastructures. These initiatives will use participatory design methods to garner community engagement through community forums and the establishment of advisory boards with local stakeholders, partnering with local communities to co-design AI tools tailored to regional healthcare needs, thereby fostering inclusivity and trust [69]. This comprehensive approach ensures that equity considerations are embedded throughout the AI lifecycle, from development to deployment and ongoing monitoring (Table 1).

**Table 1:** Core components of the comprehensive algorithmic oversight and stewardship (CAOS) framework for healthcare AI

Component	Purpose	Key Features	Implementation Considerations	Resource Requirements	Interdependencies with Other Components
**Stratified Algorithmic Risk Assessment Protocol (SARAP)**	Categorize AI systems by potential risk to patient outcomes, data sensitivity, and operational dependency.	3D risk scoring system, normalized Euclidean distance, tiered oversight (0–4).	Requires integration with AI governance policies and continuous monitoring of AI risk scores.	Computational infrastructure for risk assessment, expert oversight, and regulatory frameworks.	Informs frequency and depth of ABEMF bias audits and retraining; dictates interpretability levels required by CICRE; guides DRAM oversight intensity.
**Quantum-Resistant Federated Learning Network (QRFLN)**	Ensures data privacy and improves AI model performance across institutions.	Lattice-based cryptography, homomorphic encryption, differential privacy, SMPC protocols.	Secure cross-institutional collaboration and compliance with privacy laws.	High-performance computing, cryptographic infrastructure, and legal support for data-sharing agreements.	Supports data infrastructure needed for ABEMF retraining and CICRE’s causal modeling using distributed datasets.
**Causal Inference and Counterfactual Reasoning Engine (CICRE)**	Provide causally grounded explanations for AI-driven decisions.	Structural causal models, do-calculus, counterfactual explanations.	Requires high-quality labeled datasets and domain expertise in causal modeling.	Advanced statistical software, expertise in causal inference, and training for AI teams.	Receives interpretability level requirements based on SARAP tiering; outputs support regulatory reporting in DRAM.
**Algorithmic Bias and Equity Mitigation Framework (ABEMF)**	Continuously monitor equity and mitigate AI biases.	Bias and Equity Evaluation Board (BEEB), equity retraining pipelines, and pre-deployment audits.	Needs continuous bias detection and retraining mechanisms and diverse training datasets.	Bias detection tools, diverse data access, dedicated equity review teams.	Retraining cadence and audit rigor are calibrated to SARAP tiers; fairness metrics inform DRAM policy updates.
**Dynamic Regulatory Adaptation Mechanism (DRAM)**	Adapt governance to emerging AI technologies.	Policy roundtables, ethical impact assessments.	Must be adaptable to new regulatory changes and involve multiple stakeholders.	Policy research teams, international regulatory liaisons, and AI ethics experts.	Uses outputs from SARAP, CICRE, and ABEMF to adjust oversight policies and trigger regulatory updates.

## Discussion

The Comprehensive Algorithmic Oversight and Stewardship (CAOS) Framework represents a foundational step toward addressing the regulatory and accountability gaps associated with shadow AI in healthcare. By introducing dynamic licensing systems, equity-centered governance, and continuous post-market monitoring, we aim to help facilitate the responsible deployment of AI technologies, with safety and equity as paramount concerns. This framework not only mitigates risks like model drift and bias but also establishes mechanisms to harmonize evolving federal, state, and international AI regulations. Potential metrics for CAOS success include reductions in AI-related adverse events, improved equity metrics across AI performance reports, and responsiveness of regulatory updates through DRAM tracking logs, offering a means to evaluate the framework’s real-world impact over time.

While the CAOS Framework offers a flexible and adaptive model for integrating innovation with accountability, its adoption may encounter resistance from various stakeholders. Developers may perceive compliance requirements as constraints on innovation, administrators might be concerned about financial and logistical burdens, and clinicians may be hesitant to integrate AI-driven decision-making into clinical workflows. To address these concerns, a phased implementation roadmap is proposed, allowing organizations to progressively adopt framework components based on capacity, system maturity, and AI risk profiles.

At a high level, this roadmap prioritizes the foundational elements that can deliver immediate safeguards with minimal infrastructural overhaul. Phase 1 involves establishing the AI Competency and Stakeholder Education Program (AI-CSEP) to build basic AI literacy and training infrastructure across roles. Concurrently, organizations can implement SARAP to assess existing AI systems using standardized, risk-based criteria. In Phase 2, the AI Liability Certification (AILC) and CICRE components should be integrated to ensure high-risk AI systems are clinically validated and interpretable. Phase 3 introduces ABEMF equity pipelines and DRAM adaptive oversight to manage performance drift and policy responsiveness. Finally, Phase 4 facilitates global compliance through the Global Alignment Network (GAN), enabling institutions to harmonize practices with international regulatory standards. This incremental approach enables smoother transitions and minimizes disruption to existing healthcare operations.

It addresses several key challenges highlighted by existing legal frameworks:

1. **Liability Distribution**: The framework’s **Stratified Algorithmic Risk Assessment Protocol** (**SARAP**) provides a mechanism for assigning proportional liability across stakeholders based on the risk tier of the AI system. This approach complements existing legal doctrines by accounting for the evolving nature of AI technologies while ensuring that accountability is distributed fairly.
2. **Clinical Validation Standards**: Within the broader CAOS Framework, the **AI Liability Certification** (AILC) establishes rigorous protocols for testing AI systems against diverse patient populations and real-world scenarios. This approach fills crucial gaps in existing oversight mechanisms, particularly those left by current regulatory frameworks, including the 21st Century Cures Act. However, implementation across resource-constrained settings may be challenging, necessitating alternative compliance pathways that balance regulatory rigor with feasibility.
3. **Regulatory Evolution**: Through its **Dynamic Regulatory Adaptation Mechanism** (**DRAM**), the framework maintains contemporary relevance by incorporating emerging technologies and shifting healthcare needs into its oversight protocols. To ensure global compatibility, the CAOS Framework aligns with international standards such as ISO/IEC AI safety guidelines and ongoing regulatory developments from entities like the European Medicines Agency (EMA) and the World Health Organization (WHO).

Even though adopting the CAOS Framework would require substantial investment and adaptation of existing processes, these efforts are essential for improving healthcare AI safety and equity. Initially, healthcare facilities, particularly smaller institutions, may face difficulties in resource allocation, funding, workflow adjustments, and structural adaptations that could slow AI adoption. However, policymakers must ensure that regulation does not stifle innovation, especially in resource-limited settings. Targeted support mechanisms, such as subsidized compliance programs and open-access regulatory toolkits, can help mitigate these barriers and enable more institutions to get access to incredible equipment that can increase mortality outcomes. Despite these initial hurdles, the framework’s capacity to enhance patient care, strengthen clinical trust, and advance responsible innovation outweighs these challenges and must be considered.

To further support adoption in under-resourced environments, core components of the CAOS Framework, such as ABEMF and EAIP, have been designed with modularity and scalability in mind. ABEMF includes lightweight bias audit templates and supports modular deployment of equity calibration pipelines, allowing institutions to prioritize essential fairness checks aligned with their data and infrastructure capacity. Similarly, the EAIP can be adapted for resource-limited settings by leveraging community partnerships, using open-access datasets, and emphasizing participatory design methods over resource-intensive algorithmic retraining. For institutions facing severe constraints, the CAOS Framework identifies a subset of “non-negotiable” tools essential for ethical AI deployment: SARAP for risk-tier assignment, minimal ABEMF audits for bias detection, and CICRE-lite modules offering simplified interpretability for high-risk systems. These stripped-down yet functional components ensure that ethical AI governance can be achieved even in the absence of advanced computing infrastructure, preserving the framework’s commitment to equity and safety across all care settings.

Ultimately, the CAOS Framework is not only a practically useful roadmap—it is an ethically necessary intervention in the face of rapidly evolving, high-impact AI technologies. By embedding core values such as trust, safety, and justice into the architecture of AI oversight, CAOS helps ensure that technological progress in healthcare serves the public good. It advances a vision of healthcare AI that is not merely innovative but also accountable, equitable, and aligned with foundational medical ethics. Looking ahead, the CAOS Framework establishes foundational guidelines for responsible AI deployment by merging technological progress with established medical ethics and regulatory standards. This creates a sustainable path for AI integration that prioritizes both innovation and patient welfare, ensuring equitable benefits across diverse healthcare landscapes.

### Acronyms

**Table:** 

Acronym	Meaning	Other Term Used
**SARAP**	Stratified Algorithmic Risk Assessment Protocol	The risk-tiering system
**QRFLN**	Quantum-Resistant Federated Learning Network	The explainability and consent framework
**CICRE**	Causal Inference and Counterfactual Reasoning Engine	The AI output explainability system
**ABEMF**	Algorithmic Bias and Equity Mitigation Framework	Bias mitigation pipeline
**DRAM**	Dynamic Regulatory Adaptation Mechanism	The dynamic regulatory update mechanism
**AI-CSEP**	AI Competency and Stakeholder Education Program	AI literacy and training module
**EAIP**	Equity-Centric AI Implementation Protocol	The ethical deployment protocol

## Electronic Supplementary Material

Below is the link to the electronic supplementary material.

Supplementary Material 1

## Data Availability

No datasets were generated or analysed during the current study.
