# Supplementary material for: Navigating Healthcare AI Governance: the Comprehensive Algorithmic Oversight and Stewardship Framework for Risk and Equity
Source: Health Care Anal. 2025 Aug 13;34(3):719–43. doi: 10.1007/s10728-025-00537-y (PMC13675346; doi:10.1007/s10728-025-00537-y)
Supplement: Supplementary file 1 [file 10728_2025_537_MOESM1_ESM.docx]

**Navigating Healthcare AI Governance: The Comprehensive Algorithmic Oversight and Stewardship Framework for Risk and Equity**

Rahul Kumar, BS^1,2^, Kyle Sporn^3^, Ethan Waisberg, MB BCh BAO^4✉^, Joshua Ong, MD^5^, Phani Paladugu MMSc^6,7^, Amar S. Vadhera, BS^6^, Dylan Amiri, BS^8^, Alex Ngo, BS^1^, Ram Jagadeesan, MS^9,10^, Alireza Tavakkoli, PhD^11^, Timothy Loftus, MD, JD^12^, Andrew G. Lee, MD^13-20^,

^1^ Department of Biochemistry and Molecular Biology, University of Miami Miller School of Medicine, Miami, Florida, United States

^2^ Batchelor Children’s Research Institute, University of Miami Miller School of Medicine, Miami, Florida, United States

^3^SUNY Upstate Medical University, Norton College of Medicine, Syracuse, NY, 13202

^4^ Department of Clinical Neurosciences, University of Cambridge, Cambridge, United Kingdom

^5^ Department of Ophthalmology and Visual Sciences, University of Michigan Kellogg Eye Center, Ann Arbor, Michigan, United States

^6^ Sidney Kimmel Medical College, Thomas Jefferson University, Philadelphia, Pennsylvania, United States

^7^ Brigham and Women’s Hospital, Harvard Medical School, Boston, Massachusetts, United States

^8^ University of Miami Miller School of Medicine, Miami, Florida, United States

^9^ Department of Computer Science, Johns Hopkins University Whiting School of Engineering, Baltimore, Maryland, United States

^10^ Artificial Intelligence Systems, Cisco Systems, Inc., San Jose, California, United States

^11^ Department of Computer Science and Engineering, University of Nevada, Reno, Reno, Nevada, United States

^12^ TL Insight US, Miami, Florida, United States

^13^ Center for Space Medicine, Baylor College of Medicine, Houston, Texas, United States

^14^ Department of Ophthalmology, Blanton Eye Institute, Houston Methodist Hospital, Houston, Texas, United States

^15^ The Houston Methodist Research Institute, Houston Methodist Hospital, Houston, Texas, United States

^16^ Departments of Ophthalmology, Neurology, and Neurosurgery, Weill Cornell Medicine, New York, New York, United States

^17^ Department of Ophthalmology, University of Texas Medical Branch, Galveston, Texas, United States

^18^ University of Texas MD Anderson Cancer Center, Houston, Texas, United States

^19^ Texas A&M College of Medicine, Texas, United States

^20^ Department of Ophthalmology, The University of Iowa Hospitals and Clinics, Iowa City, Iowa, United States

**Corresponding Author:**

Ethan Waisberg ✉

Email: cambridgemlgroup@gmail.com

University of Cambridge, Cambridge, United Kingdom

ORCID: 0000-0001-8999-0212

**Funding:** None.

**Keywords**: Healthcare Oversight, Machine Learning Risks, Safety Standards, equity in AI, Ethical Algorithms, Algorithmic Accountability.

**Author Contributions:**

RK: Conceptualized the CAOS framework, drafted the initial manuscript, and critically revised the content.

EW: Contributed to framework development, wrote sections on regulatory challenges, and edited the manuscript.

JO: Provided clinical perspective on AI implementation risks and reviewed the manuscript.

PP: Assisted with literature review, contributed to writing the introduction and discussion sections.

ASV: Helped develop the SARAP component and wrote related sections of the manuscript.

DA: Contributed to writing sections on shadow AI and regulatory gaps.

AN: Assisted with manuscript preparation and editing.

RJ: Provided technical expertise on AI systems, contributed to QRFLN section.

AT: Offered insights on AI ethics and governance, reviewed and edited the manuscript.

TL: Provided legal perspective on regulatory frameworks.

AGL: Supervised the project, provided critical revisions, and approved the final manuscript.

KS: Edited the manuscript, worked on the revisions, and wrote related sections of the manuscript

All authors reviewed and approved the final version of the manuscript and agree to be accountable for all aspects of the work.

**Competing Interests**: Dr. Andrew Lee: Amgen (speaker), Alexion (speaker); consultant Virdian, Ethyreal, Catalyst, AstraZeneca, Bristol Myers, Squibb, Stoke, US Department of Justice. Ram Jagadeesan: Cisco (Employee, Stock).

**Acknowledgments:** None
